# Implementation of a group-based lifestyle intervention programme (Healthy Heart) in general practices in The Netherlands: a mixed-methods study

**DOI:** 10.3399/BJGPO.2023.0064

**Published:** 2023-10-04

**Authors:** Anne K Smit, Rimke C Vos, Rozemarijn W Bijl, Karin J G Busch, Sanne M Verkleij, Jessica C Kiefte-de Jong, Mattijs E Numans, Tobias N Bonten

**Affiliations:** 1 Department of Public Health and Primary Care (V-0-P), Leiden University Medical Center, Leiden, The Netherlands; 2 Primary Care Group The Hague (Hadoks), Hague, The Netherlands

**Keywords:** mixed methods, general practice, lifestyle intervention programme, life style, implementation, group-based intervention, cardiovascular diseases

## Abstract

**Background:**

Lifestyle intervention programmes target behavioural risk factors that contribute to cardiovascular diseases (CVDs). Unfortunately, sustainable implementation of these programmes can be challenging. Gaining insights into the barriers and facilitators for successful implementation is important for maximising public health impact of these interventions. The Healthy Heart (HH) programme is an example of a combined lifestyle intervention programme.

**Aim:**

To analyse the reach, adoption, and implementation of the HH programme.

**Design & setting:**

A mixed-methods study conducted in a general practice setting in The Netherlands.

**Method:**

Quantitative data were collected from the Healthy Heart study (HH study), a non-randomised cluster stepped-wedge trial to assess the effect of the HH programme on patients at high risk of developing CVDs at practice level. Qualitative data were obtained through focus groups.

**Results:**

Out of 73 approached general practices, 55 implemented the HH programme. A total of 1082 patients agreed to participate in the HH study, of whom 64 patients were referred to the HH programme and 41 patients participated. Several barriers for participation were identified such as time investment, lack of risk perception, and being confident in changing lifestyle on their own. Important barriers for healthcare providers (HCPs) to refer a patient were time investment, lack of information to sufficiently inform patients, and preconceived notions regarding which patients the programme was suitable for.

**Conclusion:**

This study has offered insights from a patient and HCP perspective regarding barriers and facilitators for implementation of the group-based lifestyle intervention programme. The identified barriers and facilitators, and the suggested improvements, can be used by others who wish to implement a similar programme.

## How this fits in

Lately, there has been an increasing focus on prevention and lifestyle interventions. Insights in the reach, adoption, and implementation of these programmes (in this study, the HH programme), besides the clinical effectiveness, are essential to maximise public health impact. In this study, frequently mentioned barriers by eligible patients for participation in a lifestyle intervention programme were as follows: the time investment; lack of risk perception; and being confident in changing their lifestyle on their own. The frequently mentioned barriers by HCPs referring patients to the programme were as follows: the time investment; lacking information to sufficiently inform patients about the programme; preconceived notions regarding which patients are suitable to follow the programme; lack of feedback regarding referred patients; and having to perform many manual tasks to refer a patient.

## Introduction

CVDs are a major contributor to disability and are the leading cause of death globally.^1^ Although the aetiology of CVD is multifactorial, the World Health Organization estimated that around 80% of the premature CVDs are preventable.^2^ The most important behavioural, and thus changeable, risk factors are unhealthy diet, physical inactivity, tobacco use, and excessive use of alcohol.^2,3^

Lifestyle intervention programmes have been introduced around the world to target these behaviours and can be both effective and cost-effective when implemented well, and could facilitate significant public health impact.^4–8^

Unfortunately, lifestyle programmes, just as other evidence-based practices, often fail to reach widespread clinical usage after the trial period ends.^9,10^ When implementing an intervention programme many challenges can arise, such as funding problems, legal aspects, or organisational challenges, which can lead to not implementing the intervention in a sustainable way.^11,12^ Previous research on the implementation of combined lifestyle interventions in primary care has showed, for example, that a lack of collaboration and insufficient available time and resources often impede successful implementation.^13–17^ To illustrate, the NHS Diabetes Prevention Programme (DPP) showed that an intensive lifestyle intervention for people at high risk decreased the incidence of type 2 diabetes by 58% compared with 31% in the metformin group during a mean intervention period of 3 years.^18^ However, when adapting the DPP to real-world settings, the results were often lower and many settings did not implement the intervention in a sustainable way.^12^ In order to increase reach and impact of these programmes in the real world, it is important to gain insights into the implementation process and adjust programmes accordingly.

In this study, the implementation of the HH programme was evaluated. The HH programme is a combined group-based lifestyle intervention programme implemented in general practice centres, in the city of The Hague, The Netherlands. No such programme was available at the time (in 2017). The aim of this study was to evaluate the reach, adoption, and the implementation process of this programme, both qualitatively and quantitatively, in order to identify factors that influenced its implementation.^19^ The lessons learnt can be valuable for the development or improvement of (lifestyle) intervention programmes.

## Method

### Design and setting

This was a mixed-methods study to assess the reach, adoption, and implementation of the HH programme.

#### The Healthy Heart programme

The HH programme was developed by the primary care cooperative organisation Hadoks, which represents and supports the vast majority of GPs in The Hague. Currently, The Hague is the third largest city in The Netherlands with 550 000 inhabitants, of whom 56.2% have a migratory background.^20^

The group-based combined lifestyle intervention programme, led by a certified lifestyle coach, was intended for patients based in The Hague with CVD or a high risk of developing CVD, type 2 diabetes, asthma, or chronic obstructive pulmonary disease (COPD). As part of usual care, general practices affiliated with Hadoks assign these patients to respective care protocols: the primary cardiovascular prevention, secondary cardiovascular prevention, diabetes, and asthma or COPD care protocol.

The HH programme consisted of eight group sessions and two individual sessions over 5 months, focusing on multiple aspects of lifestyle change. Patients were guided to set realistic, individual goals. Patients included in one of the care protocols could be referred to the programme by their GP or practice nurse (PN).

#### The Healthy Heart study

Study design and rationale of the HH study have been described elsewhere.^21^ In short, the HH study is a non-randomised cluster stepped-wedge trial to assess the effect of the HH programme on patients in the primary cardiovascular prevention care protocol at practice level. Therefore, these patients were at high risk for developing CVD (defined as having a 10-year cardiovascular risk of ≥10%, according to Dutch cardiovascular risk management guideline, at the moment of inclusion [2017–2019]).^22^ During both the control and the intervention period, patients were invited to be included in the HH study. Being included in the study meant patients would fill in questionnaires at baseline, 3 months, 6 months, 12 months, and 24 months. Patients were also given the option to only provide informed consent for using data from their GP record. During the control period, practices offered usual care; during the intervention period patients could choose to follow the HH programme or usual care.

### Quantitative data: RE-AIM

The RE-AIM framework was used for presenting the quantitative data.^19^ This dissemination and implementation framework includes the following five dimensions: reach (R); effectiveness (E); adoption (A); implementation (I); and maintenance (M). It focuses on both individual and organisational levels in order to provide evidence on public health impact.^19^ In the current study, the focus was on reach (the proportion of eligible patients in The Hague who participated in the HH programme), adoption (the amount of general practices that implemented the HH programme), and implementation (qualitatively assessed using the Consolidated Framework of Implementation Research [CFIR], described below). Effectiveness, maintenance, and cost-effectiveness are discussed in a separate article.^23^

### Qualitative data: experiences of healthcare providers and eligible patients

Both general practices and eligible patients were selected by purposive sampling. Both patients who participated (henceforth referred to as 'participants') and patients who did not participate (henceforth referred to as 'non-participants') in the HH programme were recruited from the HH study. All the included patients were included in the HH study during the intervention period, so they should have been offered the HH programme by the HCP.

One focus group per general practice was organised. The interview guide (provided in Supplementary Box S1) was composed using the CFIR, a conceptual framework containing 39 constructs arranged across five domains (intervention characteristics, outer setting, inner setting, characteristics of individuals, process), which are likely to influence the implementation of complex programmes.^24^

For the patients, focus groups were organised separately for participants and non-participants. The interview guide is provided in Supplementary Box S2.

All interviews were audio-recorded and transcribed verbatim. Focus groups were organised until data saturation was achieved.^25^

### Coding and analysis

For analysing the experiences of the HCPs the framework method was used to facilitate the coding and the analysis process.^26^ CFIR constructs were coded deductively.^27^ For the experiences of the patients, barriers and facilitators for joining the programme and experiences with the programme were coded inductively and grouped into themes using a thematic analysis.^28^

Two researchers (AS and RB) independently coded two transcripts of the focus groups with HCPs — one transcript of a focus group with participants and one with non-participants — using ATLAS-ti (version 7). Afterwards, for the focus groups with HCPs, codes were compared and, in case of disagreement, a discussion took place and the codebook was updated. It was possible to resolve all disagreements this way. For the focus groups with (non-)participants, labels were compared and the researchers agreed on a set of codes. After this, AS continued to code the remaining transcripts using the updated codebook. A discussion with RB took place when new codes emerged.

## Results

### Quantitative results

#### Reach and adoption

In total, 73 general practices were approached to adopt the HH programme, of which 55 practices (75%) agreed to participate. Reasons for not adopting the programme were as follows: absence of PN (*n* = 4); burden on PN (*n* =1); lack of time (*n* = 1); and no longer connected to the overarching primary care group (*n* = 1). The remaining 11 practices did not state reasons for non-participation.

**Figure 1.**
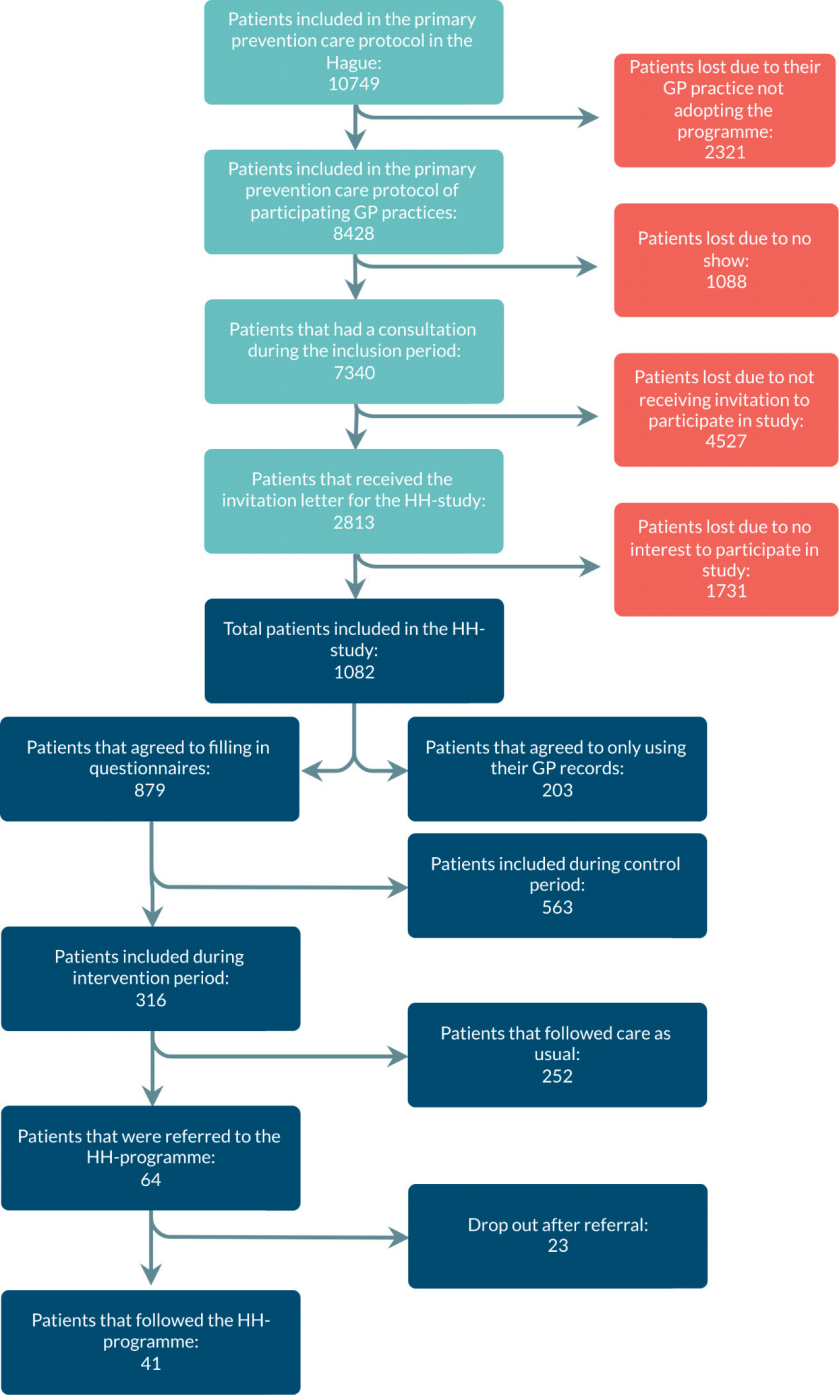
Flowchart of referrals to the Healthy Heart programme

The total number of patients in the primary prevention care protocol of all the approached practices was 10 749 at the start of the programme. The 55 practices that adopted the programme took care of 8428 (78%) patients in this protocol, and 7340 (68%; percentages are out of the total population of 10 749) had a consultation during the inclusion period of the study. Of these, 2813 (26%) received the invitation letter and 1082 (10%) agreed to participate in the HH study. Of these, 879 (8%) agreed on filling in the questionnaires (the other 203 individuals only agreed to the use of their GP record data). Out of the 879 participants, 316 (2.9%) were included during the intervention period and should have been offered the HH programme. Of these 316 patients, 64 patients were actually referred to the HH programme and 41 patients eventually participated (see Figure 1). The 23 patients who were referred to the HH programme but did not participate either did not attend the intake or decided (together with the lifestyle coach) that the programme was not suitable for them.

In total, 262 of the 316 patients (37 participants and 225 non-participants of the HH programme) included during the intervention period, filled in the baseline questionnaire. Characteristics can be found in Table 1. Participants of the HH programme were on average younger; were more often female; more often had a migratory background; more often had a lower educational level; had a higher weight and body mass index (BMI) at baseline; and more often had the goal to lose weight, increase physical activity, and maintain a healthier diet.

**Table 1.** Baseline characteristics of participants and non-participants of the Healthy Heart programme

Characteristic	Participants lifestyle programme (*n* = 37)	Non-participants lifestyle programme (*n* = 225)	*P value*
Mean age, years (SD)	59.7 (10.21)	65.2 (9.77)	0.002^a^
Female gender, *n* (%)	27 (73.0%)	123 (54.7%)	0.037^a^
Migratory background, *n* (%)	13 (35.1%)	36 (16.0%)	0.006^a^
Educational level, *n* (%)			0.025^a^
Lower	13 (36.1%)	37 (16.8%)	
Middle	11 (30.6%)	90 (40.9%)	
Higher	12 (33.3%)	93 (42.3%)	
Employed (aged <65 years), *n* (%)	16 (64.0%)	65 (67.0%)	0.776
Mean weight, kg (SD)	94.4 (24.0)	80.5 (14.8)	0.002^a^
Mean BMI, kg/m^2^ (SD)	32.3 (6.6)	27.4 (5.73)	0.000^a^
Mean waist circumference, cm (SD)	109.5 (13,6)	98.2 (11,9)	0.000^a^
Medication, *n* (%)			
Antihypertensive	29 (80.6%)	164 (74.5%)	0.438
Cholesterol-lowering	18 (50.0%)	104 (47.3%)	0.761
Smoking in past 6 months, *n* (%)	5 (13.9%)	20 (9.1%)	0.374
Mean alcohol consumption, glasses/week (SD)	6.1 (9.3)	6.9 (8.0)	0.553
Hospital admission in past 6 months, *n* (%)	1 (2.8%)	11 (5.0%)	0.559
Mean General self-efficacy (SD)^b^	32.9 (4.4)	32.7 (5.3)	0.831
Lifestyle goals at baseline, *n* (%)			
Weight loss	34 (91.9%)	147 (65.3%)	0.001^a^
Increasing physical activity	32 (86.5%)	140 (62.2%)	0.004^a^
Healthier diet	32 (86.5%)	130 (57.8%)	0.001^a^
Quit smoking	1 (2.7%)	5 (2.2%)	0.603
Reducing alcohol intake	8 (28.6%)	51 (30.7%)	0.819

^a^Significant result. ^b^Measured with the General Self-Efficacy Scale. BMI = body mass index.

Missing data for participants of programme: Eductional level: 1 missing; Employed: 12 missing (mostly due to being over 65 years of age); mean weight: 1 missing; Mean BMI: 1 missing; Mean waist circumference: 2 missing; Medication: 1 missing; Smoking: 1 missing; Alcohol: 2 missing; Hospital admission: 1 missing; General self efficacy: 1 missing; Reduce alcohol intake: 9 missing. Missing data for non-participants of programme: Mean age: 1 missing; BMI: 2 missing; Waist circumference: 8 missing; Educational level: 5 missing; Employed: 128 missing; Medication: 5 missing; Smoking: 5 missing; Mean alcohol: 6 missing; Hospital admission: 5 missing; General self-efficacy: 6 missing; Reducing alcohol intake: 51 missing.

### Qualitative results

In total, five general practices (including 12 PNs and five GPs) were included in the focus groups. One practice was a smaller general practice, the other four were part of larger healthcare centres.

In total, 31 eligible patients participated in the focus groups, of whom 10 were participants and 21 were non-participants of the HH programme. The (non-)participants were representative of the study population described in Table 1.

**Figure 2.**
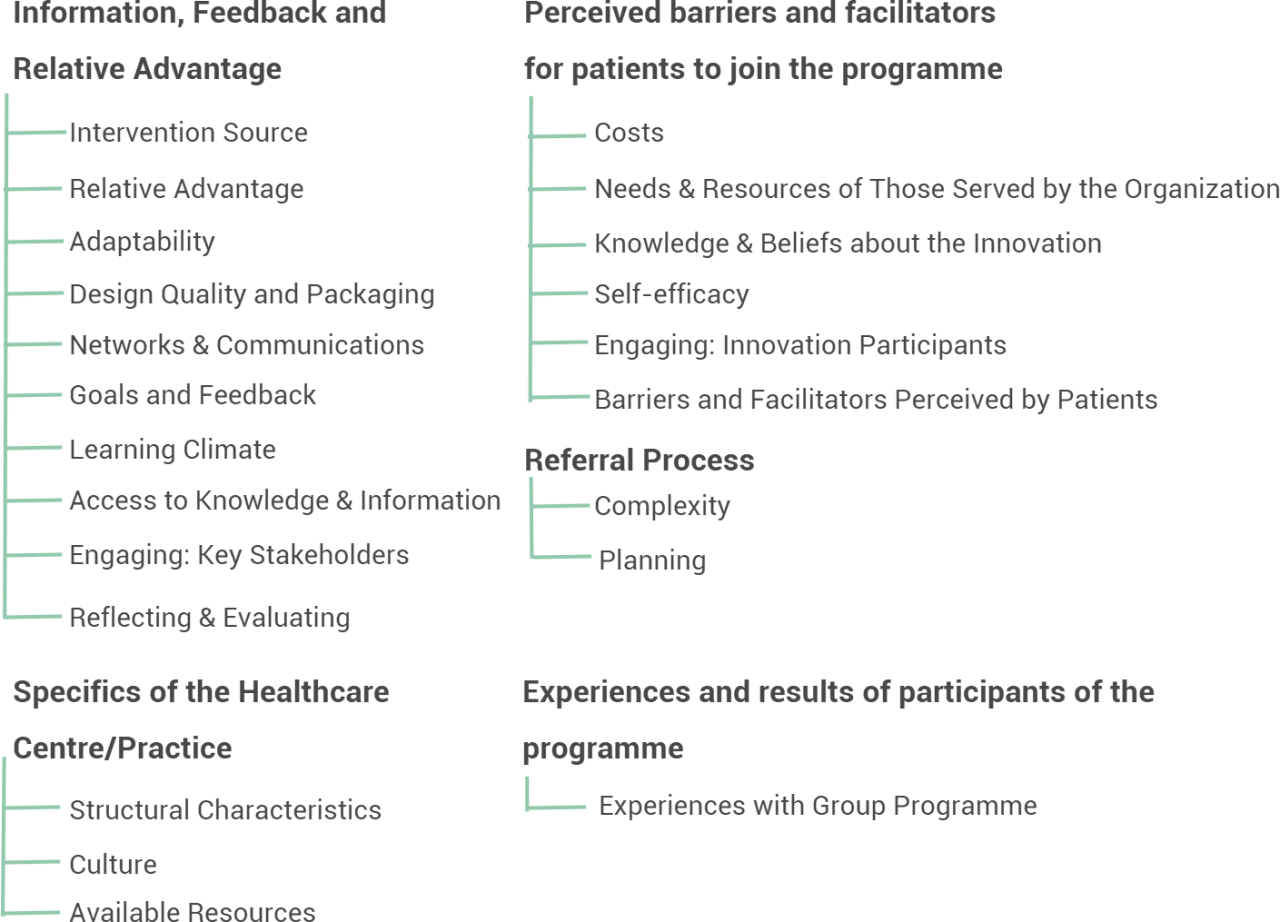
Identified themes with associated Consolidated Framework for Implementation Research constructs and sub-themes

Of the 36 CFIR constructs, 20 were discussed during the focus groups with the HCPs. Together with the identified barriers and facilitators, these constructs were grouped into the following four main themes: information, feedback, and relative advantage; perceived barriers and facilitators for patients to join the programme; referral process; and specifics of the healthcare centre or practice. The experiences of the participants with the programme formed a fifth theme. See Figure 2 for an overview of the themes and associated CFIR constructs and sub-themes.

#### Information, feedback, and relative advantage

Mentioned barriers and suggested improvements by HCPs for referring patients to the programme are summarised in Table 2. Most HCPs mentioned that the information about the organisation and design of the programme was clear but information regarding the content was lacking. This made it hard to ‘sell’ the programme to the patients:

**Table 2.** Mentioned barriers for referral of eligible patients, as mentioned by the HCPs, and possible improvements

Theme	Barriers for referral	Possible improvements
Information, feedback, and relative advantage	Lack of information regarding content of the programme, which makes it hard to 'sell' the programme to the patients Lack of feedback from referred patients Lack of information and feedback may result in not seeing the relative advantage over other options	Offer sufficient information about the programme; for example, flyers, a kick-off meeting Offer the HCP the option to experience a session Offer feedback from participants in order to keep HCPs involved; for example, through a newsletter, a dashboard, a report about the results of all the participants Involve HCPs in development of the programme in order to keep them engaged Appoint a local site leader in every general practice
Perceived barriers and facilitators for patients to join the programme	Preconceived notions about which patients are suitable for the programme Preconceived notions about what patients want in a programme	Offer feedback from participants in order to inform HCPs about how the programme is experienced by patients in order to reduce preconceived notions
Referral process	Many manual tasks to refer a patient Lack of clarity about starting dates makes it hard to inform the patients	Reduce amount of manual tasks for HCP Appoint a programme leader to handle procedural tasks, do the planning, and serve as a central point of contact for questions about the programme
Specifics of the healthcare centre or practice	Lack of time to inform a patient about the programme	Offer informative flyers, booklets and a website to which the HCP can refer a patient for more information about the programme

HCP = healthcare provider

*'You cannot sell a car that you have not driven yourself.'* (Practice nurse)

One PN asked if she could attend one session; however, this was declined because the lifestyle coach was concerned that her presence might influence the group dynamics

Moreover, HCPs mentioned that they would have liked to get more detailed feedback on the progress of the referred patients instead of receiving minor feedback via a digital information portal. The relationship with the lifestyle coach also seemed to influence this. Three out of the five practices mentioned that the communication with the lifestyle coach went smoothly through email or by phone. Good communication resulted in the HCPs feeling more involved in the programme:

*'That* [the communication] *goes well, which is nice because she* [the lifestyle coach] *always updates you and that gives me the feeling I am still involved.'* (Practice nurse)

One practice was very positive about the programme and thought that addressing stress alongside diet and exercise gave the programme an advantage over other options. Two of the practices explicitly mentioned that they would like to receive more feedback and information about the programme before they could decide on the advantage. One practice was particularly negative about the programme; PNs from this practice observed that it was easier to refer patients to a dietician they already had good experiences with. Also, they felt that there were already a great number of lifestyle options available and that new (external) projects kept pouring in and this cost them lots of energy. This seemed to influence the motivation of the PNs to inform themselves about this programme, and may also have been the reason that their feedback only focused on a small section of the programme (the visit to the supermarket):

*'If I had to go myself, I wouldn’t want to walk into a supermarket with a group of people and read labels. I wouldn’t feel like it at all.'* (Practice nurse)

#### Perceived barriers and facilitators for patients to join the programme

The HCPs mentioned several facilitators and barriers for eligible patients to join the programme. Some of them they learnt through feedback; however, some seemed to stem from preconceived notions about which patients would benefit from the programme. In Table 3 the mentioned facilitators and barriers by the HCPs are summarised and compared with those mentioned by the patients.

**Table 3.** Perceived barriers and facilitators of patients for joining the programme as mentioned by healthcare providers, participants, and non-participants of the lifestyle programme

	Barriers	Facilitators
**Healthcare providers (*n* = 15**)	Group (*n* = 10) Time investment (*n* = 10) Not worried about current lifestyle or health (*n* = 8) Not seeing relative advantage over other options (going to the gym, dietician) (*n* = 3) Language and literacy (*n* = 2) Location in neighbourhood (*n* = 1)	Free of charge (*n* = 9) Location in neighbourhood (*n* = 7) Worried about abnormal blood values, experiencing symptoms, or having to start medication (*n* = 7) Eager to gain new knowledge (*n* = 3)
**Participants lifestyle programme (*n* = 10**)	Time investment (*n* = 1)	Having abnormal blood values, experiencing symptoms, or having to start medication (*n* = 2) Group (*n* = 2) Eager to gain new knowledge (*n* = 1) Location in neighbourhood (*n* = 2) Free of charge (*n* = 1)
**Non-participants lifestyle programme (*n* = 21**)	Not being informed about the existence of the programme (*n* = 14) Confident in changing lifestyle on their own (*n* = 11) Time investment (*n* = 9) Not worried about current lifestyle or health (*n* = 6) Not seeing relative advantage over other options (going to the gym, dietician) (*n* = 5) Group (*n* = 1) Worried the programme might be too intensive owing to experienced health problems (*n* = 1)	Eager to gain new knowledge (*n* = 5) Location in neighbourhood (*n* = 5) Group (*n* = 4) Worried about abnormal blood values, experiencing symptoms or having to start medication (*n* = 3) Free of charge (*n* = 3) More accessible for people with health problems (*n* = 2)

Interestingly, many non-participants had no knowledge about the existence of the programme, which could be partly owing to the fact that HCPs had different beliefs about which patients the programme was suitable for. On the one hand, it was mentioned that the programme should not target patients with chronic diseases because they will already have received information through other sources. On the other hand, some mentioned they did not refer patients who only had hypertension since these patients do not need such an intensive programme. One PN mentioned she only referred patients who, in her opinion, were doing things completely wrong.

HCPs perceived the group-based aspect of the programme as a large barrier for patients to participate. Interestingly, most patients did not express particular concern on the fact that the programme was group-based and for some it was even a facilitator. They thought being in a group with others might give them new insights, and meeting new people was important to some:

*'It* [changing your lifestyle] *is a lonely fight with yourself, and I thought if I could share that* [with others] *… That might help.'* (Programme participant)

Time investment was mentioned as a barrier for patients to join the programme by both the HCPs and patients. This was often mentioned by patients who had a job, especially a full-time job.

Another frequently mentioned barrier by HCPs was that some patients had a lack of risk perception regarding their current lifestyle. This was also mentioned by some of the patients. In addition, some eligible patients mentioned they felt confident changing their lifestyle without any professional help.

A clear facilitator, mentioned by the HCPs, was that the programme was free and located in the neighbourhood. Some eligible patients agreed on the location and the fact that the programme was free of charge as being a facilitator; however, others mentioned they would not mind travelling to the programme or paying a small fee.

*'We want to do everything for our health, except if we have to pay 10 cents, then they* [the patients] *won’t do it.'* (Practice nurse)

All HCPs expressed they did not find it hard to discuss lifestyle with their patients, but that it was easier to motivate patients to join the programme when they experienced symptoms, had abnormal blood values, or had to start medication. Some eligible patients also mentioned this as a facilitator for joining the programme.

#### Referral process

All but one of the practices mentioned that the referral process did not always run smoothly.

When groups were full, new participants sometimes had to wait for a long time before a new group started. The fact that the dates and times were not set beforehand made it hard to inform the patients and was a reason to refer to other options.

Also, HCPs sometimes had to manually find out which lifestyle coach they should refer to at that moment. When they referred to a coach who had already started a group, they had to remove the digital referral and then refer again to another lifestyle coach. Referring to a central point was mentioned as a potential improvement.

#### Specifics of the healthcare centre or practice

One of the PNs working at the smaller practice had more flexibility to schedule her own appointments and reserve extra time for a consultation when necessary. Mainly the PNs working at the larger healthcare centres explained that finding time to inform patients about the lifestyle programme was a challenge, which could have resulted in a lower participation rate.

#### Experiences of participants

Overall, participants were very positive about the group-based character of the programme. One participant mentioned that a fellow participant in the group had asked them to join their gymnastics club, something they would never have done on their own. There were a few downsides mentioned to the fact that the programme was group-based. The advantage of a diverse group was that patients received different views on certain topics, but this could also make it hard to feel a connection with the other participants. This was especially mentioned by participants who joined the group programme because they wanted to make new acquaintances. Lastly, one participant mentioned that the group dynamics in their group were disrupted because often people did not show up to the meeting.

## Discussion

### Summary

The aim of this study was to analyse the reach, adoption, and implementation of the HH programme. The adoption rate of the HH programme was 75%. In this study, 0.6% (64 patients) of the target population of 10 749 (individuals included in the primary prevention care protocol in the Hague) was referred to the HH programme. Because of the stepped-wedge design of the HH study, not all patients had a chance to be referred to the HH programme. As a result, of the 316 patients included in the HH study during the intervention period, 64 patients (20%) were referred to the HH programme.

Frequently mentioned barriers perceived by eligible patients were the time investment, being confident in changing lifestyle on their own, and a lack of risk perception.

Frequently mentioned barriers for referral of patients by HCPs were as follows: lack of information about the content of the programme, which made it hard to 'sell' the programme to the patient; lack of feedback from referred patients, which decreased the engagement of the HCP with the programme; lack of time to inform and refer patients; a complicated referral system; and, lastly, preconceived notions about what they believed patients wanted in a programme.

### Strength and limitations

A strength of this study is the mixed-methods design. The quantitative data gave information about the amount of referred patients and their characteristics. The qualitative findings offered more insight into factors that might have contributed to these numbers. Also, because the interviews included different stakeholders, it was possible to compare ideas about the programme.

In interpreting the results of this study, some limitations should be considered. An important limitation was that it was possible for patients from the primary prevention protocol to be referred to the HH programme but not be included in the HH study. The differences in characteristics of the study group might therefore be biased by the reach of the HH study rather than only the reach of the HH programme, and the reach illustrated in this study could be an underestimation. Also the stepped-wedge design of the HH study influenced the reach because patients included during the control period could not be referred to the HH programme. Another complicating factor to analyse the reach of the HH programme was that a similar combined lifestyle intervention programme was simultaneously implemented and promoted during the study period. Possibly, patients who met inclusion criteria for the HH programme might have been referred to this alternative. Second, the programme was also intended for patients from other care protocols; however, data about these patients was lacking because they were not included in the HH study. It would have been interesting to compare the number of referred individuals between the groups. Lastly, because the study used interviews, participants might have given socially acceptable answers, especially because patient participants were interviewed in focus groups. The research team attempted to limit such bias by emphasising that answers would be handled anonymously.

### Comparison with existing literature

Not reaching the complete target population can be partly explained by the fact that approximately 25% of practices did not adopt the programme. The main reason stated for this was the lack of available PNs, illustrating that lifestyle counselling is mostly performed by PNs rather than GPs.

Also, the previously mentioned barriers by patients influenced the reach of the programme. One of the barriers was the lack of risk perception, this might especially be the case in the primary cardiovascular prevention subgroup, since these patients probably experience fewer symptoms, might have experienced fewer teachable moments, and could therefore have a lower risk perception than those with a diagnosed chronic condition. Teachable moments are often defined as changes in life or health events (such as new onset of diabetes, pain in joints, abnormal test results, and hospitalisation), which could motivate an individual to change their lifestyle.^29,30^ Moreover, the patients who did participate in the HH programme had on average a higher weight, BMI, waist circumference, and more often had the goal to lose weight, increase physical activity, and eat healthier. Therefore, they might have been more motivated to join the programme. The goal of losing weight as the most important motivator for participating in a lifestyle intervention programme has also been described previously.^17^

A surprising finding was that participants of the HH programme more often had a migratory background and a lower educational level. Often, people with a low socioeconomic status (SES) are reached less by health-promoting interventions, which increases socioeconomic health inequalities.^31,32^ The fact that the programme was partially personalised might have made it more suitable for patients with a low SES, as they more often experience material and psychosocial problems.^31^ Previous work has shown that the deployment of lifestyle coaches with an ethnically matched background can increase the reach to patients with a migratory background.^33^

Remarkably, the most prevalent mentioned barrier was that patients were not informed about the existence of the programme. This also puts the results of the effectiveness and cost-effectiveness study in a different light;^23^ this study investigated whether implementing the HH programme was effective and cost-effective on a population level by giving lifestyle change a boost during the intervention period. Implementation of the programme in general practices was found not to be effective or cost-effective on a population level. However, the results from the current study showed that also in the intervention period, most patients were not exposed to the new option of being referred to a lifestyle programme, which could explain why implementing the intervention was not effective or cost-effective on population level.

To explain why not all patients were exposed to the programme, the experienced barriers for referral mentioned by the HCPs,should be addressed. The barriers mentioned in this study have also been identified in previous research.^13,14,16^ When designing a similar programme, it is important to realise in advance that factors, such as little time, are common in general practice and adjust the programme accordingly.

HCPs had specific thoughts about which patients were suitable for the programme. They may have made an initial selection of patients they considered to have the right motivation and capacities to participate, and therefore did not offer the programme to everyone. As described earlier, it could be useful for recruitment to use less strict inclusion criteria and to give the HCP more freedom in selection for referral.^33^ However, the present study has shown that there is a risk that biases, implicit or otherwise, could lead to the programme not being offered to patients who could be suitable and this could lead to a lower reach.

It is especially interesting that the HCPs mentioned that they thought the information about the content of the HH programme was scarce, given that much energy was put into informing the HCPs by organising a large kick-off meeting and creating informative flyers for patients and HCPs. The question remains whether the offered information was unclear, or the information did not reach the HCPs. In case of the latter, there could be multiple causes such as HCPs not being able to attend the kick-off meeting or not being willing or able to actively inform themselves about the programme. The lack of awareness about specifics of the programme was also found in a study evaluating the implementation of the NHS DPP in England.^34^ Appointing one of the HCPs in a practice as a local site leader may help in keeping others working in the practice informed and engaged.

Interestingly, the HCPs thought the fact that the programme was a group-based programme was a large barrier for patients to join the programme. However, most participants and non-participants did not express particular concern around the fact that the programme was group-based, and for some it was even a facilitator. Giving feedback on this to the HCPs might have made them more comfortable in offering the programme to patients they thought would not want to join a group programme. Participants of the programme were mostly positive about the group-based character of the programme. This has also been illustrated in other studies about group programmes.^35,36^

### Implications for practice

In this article, the reach, adoption, and implementation of the HH programme were assessed. Insights from a patient and HCP perspective are provided regarding the implementation of the group-based lifestyle intervention programme.

HCPs mentioned that the time investment was a large barrier for offering the programme to patients. Offering sufficient information materials (for example, flyers, a website) was mentioned as a solution to decrease the amount of time the HCP has to invest in informing the patients. To save time, the referral process should run smoothly; for example, through an electronic referral system in order to limit procedural tasks undertaken by the HCP.

Also, time should be invested in keeping the HCPs engaged; for example, by involvement of HCPs in the development of the programme, appointing a local site leader in every general practice, and offering regular feedback from participants to the HCPs.

Furthermore, HCPs had preconceived notions about which patients were suitable for the programme; however, these did not always match with the patients’ views. Giving regular feedback from referred patients to the HCPs could keep them engaged and reduce these preconceived notions.

Mentioned barriers by patients for participation in the programme were the time investment, lack of risk perception, and being confident in changing lifestyle on their own. Addressing these barriers during the consultation with the HCP could possibly motivate them to join the programme. Also sharing experiences from participants to whom the patient can relate could help increase motivation.

## Data

The dataset relied on in this article is available from the corresponding author on reasonable request.
